# Metabolic Network Discovery by Top-Down and Bottom-Up Approaches and Paths for Reconciliation

**DOI:** 10.3389/fbioe.2014.00062

**Published:** 2014-12-03

**Authors:** Tunahan Çakır, Mohammad Jafar Khatibipour

**Affiliations:** ^1^Computational Systems Biology Group, Department of Bioengineering, Gebze Technical University (formerly known as Gebze Institute of Technology), Gebze, Turkey; ^2^Department of Chemical Engineering, Gebze Technical University (formerly known as Gebze Institute of Technology), Gebze, Turkey

**Keywords:** constraint-based models, metabolic network inference, active metabolic state, metabolome, network biology, reverse engineering, flux-balance analysis

## Abstract

The primary focus in the network-centric analysis of cellular metabolism by systems biology approaches is to identify the active metabolic network for the condition of interest. Two major approaches are available for the discovery of the condition-specific metabolic networks. One approach starts from genome-scale metabolic networks, which cover all possible reactions known to occur in the related organism in a condition-independent manner, and applies methods such as the optimization-based Flux-Balance Analysis to elucidate the active network. The other approach starts from the condition-specific metabolome data, and processes the data with statistical or optimization-based methods to extract information content of the data such that the active network is inferred. These approaches, termed bottom-up and top-down, respectively, are currently employed independently. However, considering that both approaches have the same goal, they can both benefit from each other paving the way for the novel integrative analysis methods of metabolome data- and flux-analysis approaches in the post-genomic era. This study reviews the strengths of constraint-based analysis and network inference methods reported in the metabolic systems biology field; then elaborates on the potential paths to reconcile the two approaches to shed better light on how the metabolism functions.

## Introduction

Metabolic network is the outmost layer of cellular activity from the genome. The genome of a cell is a comprehensive and condensed information base, defining a boundary for the biochemical capacity of the cell. The processing of genetic information passes through several layers of fabrication and regulation before reaching their end products. This is from information to the function, from genotype to phenotype. Metabolic enzymes count for a significant percentage of the end products of genes, and their activity sets the physiology of the cell. Since metabolic network activity is the major representative of cell functionality, it is of great importance to gain as much knowledge as possible on the active metabolic network at a specific cellular state.

Systems-based approach to molecular biology has contributed to an increased knowledge of metabolic pathways for an increasing number of organisms, and led to almost complete metabolic networks for a number of major organisms, from yeast to human. Such static networks are available in a condition-independent manner through web-based databases such as KEGG or MetaCyc (Altman et al., 2013), or reconstructed in a format suitable for simulation by several researchers at genome scale (Oberhardt et al., 2009; Kim et al., 2012). There are several mathematical approaches to process such networks to come up with condition-specific networks, the most common one being the Flux-Balance Analysis (FBA) framework (Orth et al., 2010). This is a bottom-up direction toward the active network since already-known “parts,” interactions, are used as inputs (Bruggeman and Westerhoff, 2007; Petranovic and Nielsen, 2008).

In parallel to the developments on the knowledge of metabolic networks, techniques to measure metabolite levels at high throughput, termed metabolomics, have arisen (Kell, 2004; Dunn et al., 2005). Quantitative or semi-quantitative metabolome data, although one of the most challenging compared to other omic sciences, have come a long way in a decade, from the detection and quantification of about 50 metabolites (Devantier et al., 2005) to more than 1000 metabolites (Psychogios et al., 2011). Metabolome data are a snapshot of the condition-specific status of the investigated organisms. Reverse-engineering metabolome data to discover the underlying network structure is the goal behind metabolic network inference approaches (Srividhya et al., 2007; Çakır et al., 2009). The information content of metabolome data is revealed by processing it with correlation or optimization-based methods (Weckwerth et al., 2004; Hendrickx et al., 2011; Öksüz et al., 2013). Such an approach to discover metabolic network structure is termed top-down approach since the parts, interactions, are not known *a priori*, and predicted from the whole set of available biomolecules (Bruggeman and Westerhoff, 2007; Petranovic and Nielsen, 2008).

**Figure 1:**
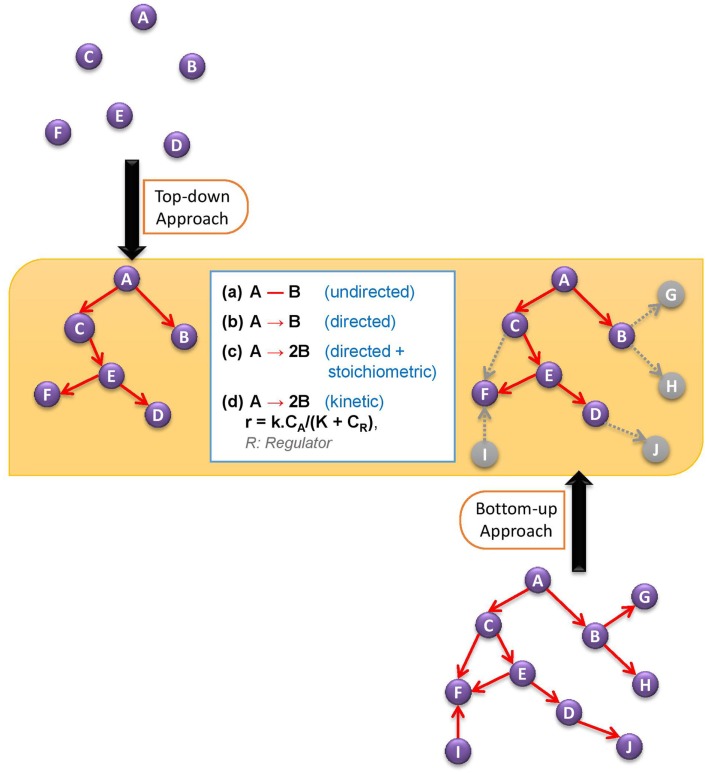
**Comparative demonstration of bottom-up and top-down approaches to discover active metabolic network**. The white box in the figure defines different levels of network structure information.

In this review, we will cover the basic developments in bottom-up and top-down approaches to discover active metabolic network, and then ponder over the possible ways of reconciling these two approaches for a better prediction of active network structure. Figure 1 illustrates the two alternative network discovery approaches.

## Bottom-Up Approaches to Discover Condition-Specific Metabolic Networks

Different methods and algorithms have been used for the discovery and characterization of active metabolic networks at different states of cells and culture environments. In the bottom-up approach, everything starts from an already available network of biochemical transformations that cover all possible scenarios in the distribution of metabolic fluxes, and sets an upper bound for the existence of reactions in the active metabolic network. Such a network is termed a static metabolic network. A static metabolic network can be provided either by a previously reconstructed genome-scale stoichiometric model or by a collection of all reactions whose existence in the organism of interest has been certified in literature and databases. Most popular among such databases are KEGG (Kanehisa et al., 2014), MetaCyc (Caspi et al., 2014), and Reactome (Croft et al., 2014). Other efforts with more curated databases such as Rhea (Alcántara et al., 2012) and MetRxn (Kumar et al., 2012) are also available. A genome-scale stoichiometric model is reconstructed based on the annotation of all genes in the genome of one organism to their end products and then to the corresponding reactions, leading to a list of gene-protein-reaction rules (Thiele and Palsson, 2010). In this way, the minimum information content of a genome-scale model is (i) a list of reactions, and (ii) a list of gene-protein-reaction rules. The presence of gene-protein-reaction rules in stoichiometric models has enabled the opportunity for transcriptome and proteome data to be incorporated into the discovery methods of active metabolic networks (Blazier and Papin, 2012).

Given a genome-scale reaction network, the aim is to find the active reaction network at a specific condition or for a specific cell type in a multicellular organism (Box 1). The core of all such discovery approaches is a stoichiometric matrix. Each row of the stoichiometric matrix represents a metabolite and each column stands for a reaction, the corresponding element being the stoichiometric coefficient of that metabolite in that reaction. The relationship between the reaction rates in the network and the dynamic change in the concentration of metabolites is represented as given below:

(1) $$\frac{d\text{C}}{\mathrm{dt}}=\text{S}\times \text{v}$$

where **S** is the stoichiometric matrix, **C** is the vector of intracellular metabolite concentrations, and **v** is a column vector of metabolic reaction rates (fluxes) to be determined. Under the assumption of steady state, the concentration of each intracellular metabolite is not going to change with time, meaning the sum of rate of reactions producing that metabolite is equivalent to the sum of rate of reactions consuming that metabolite (metabolic fluxes around each metabolite are balanced). This is represented mathematically as follows:

(2) S×v=0

This is an algebraic system of linear equations with all fluxes being zero as a trivial solution. In order to escape from the trivial solution, the value of at least one of the fluxes must be set to a non-zero value, that flux usually being an exchange flux between the intracellular and extracellular environment since the experimental measurement of exchange fluxes is relatively easier. The system is almost always underdetermined with a large solution space, mainly because of the existence of branch points in the metabolic network. There are both experimental and computational approaches to estimate a condition-specific network for such a system.

Box 1 **Different levels of Metabolic Network Structure Information.**

Our understanding of an active metabolic network can be sorted into several stages of information.

(i) At the lowest level of information, we want to know what the structure of the network is, representing it with an undirected (or directed, if the reversibility information is available) graph in which each node stands for a metabolite and each edge stands for a biochemical transformation. Alternative to the retrieval from the metabolic reaction databases, the structure of the network – both directed and undirected – can also be estimated to some extent by analyzing and reverse engineering the metabolome data without the use of *a priori* database information on the reactions.
(ii) At a higher level, the information on the stoichiometry of reactions can be incorporated, leading to a directed stoichiometric biochemical network.
(iii) Having the stoichiometric structure of the network, we can characterize the metabolic state in more detail by quantifying the metabolic fluxes. In most cases, rather than a unique flux distribution, constraints are set on flux values to shrink the solution space. Such modeling approaches are known as Constraint-Based Modeling. This level of understanding the active metabolic network (structure + flux distribution) has been the area of focus in the research community for more than a decade. In most cases, the information provided at this level has been satisfactory for engineering research to design more efficient cell factories, and also, recently, for medical research to distinguish significant differences between healthy and disease states.
(iv) There are, however, certain limitations at the above level although it provides a network activity structure weighted with fluxes. The dynamic behavior of the system cannot be captured, and the predictability power of such models is hampered mainly because they are not considering the role of regulatory mechanisms in controlling the rate of biochemical reactions. In some cases, the regulation of reaction rates plays such a dominant role that it would be hard to make any prediction by just considering the flux-based network activity structure. Here come the kinetic models into the picture, which take enzymatic regulations and metabolite concentrations into account for a dynamic and better prediction of network structure.

The experimental approach is based on stable-isotope (mostly 13C carbon) labeling of the major carbon source, and then tracing the propagation of the labeled carbon atoms down to protein-bound amino acids at isotopic steady state by using mass spectrometry or NMR spectroscopy (Wiechert et al., 2001; Sauer, 2006; Mueller and Heinzle, 2013). The qualitative isotopic labeling information is then used as an input to two alternative methods. In one method, termed isotopomer modeling, a total flux distribution is estimated based on the experimental labeling results through a computationally demanding non-linear optimization formulation, which employs global iterative fitting and statistical analysis (Wiechert et al., 2001; Antoniewicz et al., 2007). The other 13C-labeling assisted method is based on the estimation of the local ratios of fluxes emerging from a branch point (Sauer, 2006; Zamboni et al., 2009) rather than the absolute quantification of all fluxes. These experimental flux split ratios can be used to shrink the solution space of Eq. 2 in a complementary flux calculation, leading to the discovery of a condition-specific network (Schuetz et al., 2007; Tarlak et al., 2014). Softwares are available for the rather sophisticated calculation of experimental fluxes (or flux ratios) from carbon labeling data for both methods (Zamboni et al., 2005; Quek et al., 2009; Weitzel et al., 2013). A new trend in this area is to collect data at the non-stationary phase of isotopic labeling rather than at the isotopic steady state, which was shown to be more informative in terms of predicting the flux-weighted active metabolic network structure (Schaub et al., 2008; Young et al., 2008; Wiechert and Nöh, 2013). Works on the tracing of intracellular metabolites rather than only 10–15 protein-bound amino acids have also appeared due to the higher coverage of metabolic pathways despite the inherent experimental difficulties in terms of higher turnover rates as well as stability issues (Van Winden et al., 2005; Toya et al., 2007; Millard et al., 2014).

The computational approach for the discovery of condition-specific metabolic network based on Eq. 2 is known as constraint-based modeling. Constraint-based modeling methods aim to shrink the solution space of the equation as much as possible by putting relevant constraints on the system. The most common method, FBA, treats the problem in Eq. 2 as an optimization problem and linear programing is applied to solve it. The stoichiometry of metabolic reactions (stoichiometric matrix), reaction directionality information, a physiologically relevant objective function, and the value of at least one of the exchange fluxes are all that are required for FBA to return a condition-specific flux distribution. The flux distribution returned by FBA is not necessarily unique, and there may be a variety of flux distributions all leading to the same optimum value of the objective function. Therefore, Flux Variability Analysis (FVA) must be used together with FBA, to determine the variability, if any, on each metabolic flux in regard to the condition of interest (Mahadevan and Schilling, 2003; Müller and Bockmayr, 2013). The maximization of biomass production has been successfully applied as a reliable objective function for FBA to predict flux distributions in a variety of microorganisms (Varma and Palsson, 1994; Feist and Palsson, 2010). In some studies, it has been hypothesized that one objective function alone may not capture the metabolic behavior of the cell comprehensively. Therefore, multi-objective optimization platforms have been designed and utilized to come up with more specific flux distributions. Several modified versions of FBA including parsimonious FBA, pFBA (Lewis et al., 2010), and flexible-optimality FBA, flexoFBA (Tarlak et al., 2014), have been developed in this manner. On the other hand, some research groups have developed methods based on the availability of additional omics data, which are discussed below. For a thorough review of a number of FBA-derived flux calculation methods, the readers are referred to Lewis et al. (2012).

### Constraints based on transcriptome or proteome data

The rate of an enzymatic reaction inside the cell is a function of several different factors, such as the concentration of substrates, products, and regulators of the enzyme and also the amount of available active enzyme for that reaction. Among these factors, the concentration of active enzymes can be related to the activity of genes through layers of transcription, translation, and post-translational modifications. Transcriptome data are much more accessible and comprehensive compared to the other omics data. Several different research groups have developed different strategies to incorporate transcriptome data into constraint-based models. The idea behind this is that the amount of mRNAs (gene activities) may be correlated with the concentration of active enzymes, and hence this can be utilized to provide additional constraints on metabolic fluxes. At the bottom line, if an enzyme coding gene is not transcribed at steady state, the corresponding reaction should be inactive at that steady state, if there is no other enzyme catalyzing that reaction. This idea was first used by Akesson et al. to set the flux values to zero for those reactions whose corresponding genes were expressed at low levels (Åkesson et al., 2004). More sophisticated and structured versions of this approach appeared later, under the names of GIMME (Becker and Palsson, 2008) and iMAT (Shlomi et al., 2008). These approaches classify some reactions as inactive reactions based on the low expression levels of their associated genes. Then, they employ a computational framework which minimizes the contradiction between the classification and an active physiological flux distribution since some of these classifications may render the flux state unrealistic (such as zero growth rate). Several other alternative methods appeared recently to incorporate transcriptome data into the prediction of active metabolic network and flux distribution. In an interesting study, for example, mRNA levels from transcriptome data were used as weights for the corresponding reactions to predict a flux distribution without using a conventional objective function such as the maximization of biomass growth (Lee et al., 2012). A recent study (Machado and Herrgård, 2014) evaluated these methods systematically for the prediction of flux distributions, and the results were compared to that of parsimonious FBA as a reference method that does not consider the transcriptome data. In general, none of the methods could significantly improve the results of pFBA and none of them outperformed the others for the tested cases (*S. cerevisiae* and *E. coli*). Instead of the prediction of flux distributions, these methods, however, may significantly help in the discovery of active metabolic networks in context/tissue-specific cells and in the conditions where a relevant objective function cannot be hypothesized.

Transcriptome data are not necessarily correlated with the rate of corresponding reactions. Inconsistency between mRNA levels and reaction rates is a result of influence of several other factors in the regulation of enzymatic reactions. Therefore, if proteome data are available, it can be used instead of transcriptome data as a better representative for the concentration of active enzymes since proteome is hierarchically closer to the enzyme states than transcriptome data. The methods that are developed to integrate transcriptome data with the FBA method can all be used for the purpose of integrating proteome data. For example, GIMMEp (Bordbar et al., 2012) is the proteome equivalent version of GIMME. Some of such integrative methods were primarily tested with proteome data. INIT (Agren et al., 2012), for example, was developed by using proteome abundance data from Human Protein Atlas database. However, it was shown that utilizing proteome data instead of transcriptome data could not improve the prediction of flux distributions for the tested cases (*S. cerevisiae* and *E. coli*) (Machado and Herrgård, 2014). In a study which used metabolome and proteome data in the flux calculation method, on the other hand, even the use of only proteome data were shown to improve the results compared to the traditional FBA (see the next section for more details) (Yizhak et al., 2010).

Substrate concentrations, the concentration of enzyme regulators, the turn over number of the catalyzing enzyme, and the concentration of the active enzyme are all playing significant roles in the determination of reaction rates, and among them only the concentration of the active enzyme may be represented by the corresponding protein or mRNA concentration. Translated proteins are not necessarily active enzymes, and they may need to undergo post-translational modifications (e.g., phosphorylation/acetylation) to become capable of catalyzing the reactions. This is one of the main reasons behind inconsistency between protein levels and reaction rates. On the other hand, the turn over number (catalytic power) of one enzyme may differ by several orders of magnitude from the turn over number of another enzyme (Hoppe, 2012). It means that although the concentration of one enzyme may be much less than the others in the network, the reaction catalyzed by that enzyme can proceed much faster than others. According to this fact, the use of the absolute concentrations of proteins or mRNAs to constrain reaction rates does not seem promising. However, the turn over number of one enzyme in an individual is an intrinsic parameter of the enzyme that does not change from one condition to another except by effective mutations that rarely occur. Because of this, the relative levels of proteins or mRNAs can be utilized to overcome the problem of big differences in turn over numbers. One steady state with available data on flux values and protein/mRNA levels can be taken as the reference state, and then the relative/differential levels of proteins/mRNAs to the reference state can be used to predict the flux distributions at the new conditions. Based on this approach, algorithms have been developed to incorporate relative/differential transcriptome data into metabolic-flux analysis, among which are MADE (Jensen and Papin, 2011) and GX-FBA (Navid and Almaas, 2012). One other main reason for the inconsistency between protein levels and reaction rates is the distribution of flux control among different layers from genotype to phenotype. Metabolic fluxes can be regulated hierarchically (through gene expression levels) or metabolically (through metabolic interactions) (Daran-Lapujade et al., 2007; Postmus et al., 2008; Nikerel et al., 2012; Chubukov et al., 2013). Use of transcriptome or proteome data will not be helpful if the metabolic fluxes are controlled at the metabolic level.

### Constraints based on metabolome data

One approach to find more specific and physiologically relevant flux distributions is to provide additional constraints by specifying the directionality of reversible reactions. This can be done by taking Gibbs free energies of metabolites into consideration. The Gibbs free energy change of a reversible biochemical transformation (one reaction or a series of reactions) determines the direction of that transformation and its departure from reversibility. The earlier studies assumed standard conditions (all metabolite concentrations were assumed to be 1 M), and did not explicitly consider metabolite concentrations in the calculation of Gibbs energy changes of reactions due to the scarcity of metabolome data (Henry et al., 2006). Recent studies, however, take the concentration of metabolites into account, when available, to perform thermodynamic-based metabolic-flux analysis, leading to more reliable predictions (Hoppe et al., 2007; Bennett et al., 2009; Soh and Hatzimanikatis, 2010; Hamilton et al., 2013).

Extracellular metabolome data can be used to constrain genome-scale metabolic models for the calculation of intracellular flux distributions by simply constraining the secretion and uptake rates of extracellular metabolites based on such data (Çakır et al., 2007; Mo et al., 2009). In a different approach, Michaelis–Menten-based kinetics was used for the estimation of reaction rates for the reactions for which appropriate intracellular metabolome (and proteome) data are available (Yizhak et al., 2010). The FBA framework was designed in such a way that the calculated fluxes are as consistent as possible with the kinetically derived reaction rates, if available. The simultaneous use of metabolome and proteome data for this purpose significantly improved the results. The use of metabolome data alone also resulted in better predictions than the traditional FBA. In a recent study, a kinetic platform was established based on Michaelis–Menten equation to bridge gene expression levels, metabolite concentrations and metabolic fluxes without requiring the knowledge of kinetic parameters (Zelezniak et al., 2014). They could show that changes in metabolite concentrations relative to a reference steady state can be predicted by their formulation that includes information on network connectivity in addition to differential mRNA expression levels. All those works utilizing kinetic information demonstrate the necessity of dynamic models for a more comprehensive analysis of metabolic networks.

Kinetic models of biochemical reactions not only provide a rational platform for omics data – especially metabolomics – to be incorporated in the estimation of metabolic fluxes but also they enable the prediction and study of the dynamics of metabolic networks far beyond the steady state (Box 1). Such models were only possible for small-scale metabolic networks until recently (Teusink et al., 2000; Chassagnole et al., 2002), since, they require detailed information on the enzyme kinetics of each individual reaction. Estimation of kinetic parameters is a major obstacle in the applicability of dynamic modeling of metabolic networks. New platforms and algorithms were established to circumvent this problem so that the estimation of explicit kinetic parameters is not a prerequisite to study the dynamic capacity and behavior of the system (Link et al., 2014). Approximative kinetic models (lin-log, power-law, mass-action) on the other hand, try to fit a standard rate expression formula to all reactions of the network to increase the range of their applicability to larger networks (Visser et al., 2004; Sorribas et al., 2007). Thanks to approximative kinetics, attempts to reconstruct large-scale kinetic metabolic models with more than 100 reactions were recently presented (Smallbone et al., 2010; Chakrabarti et al., 2013; Stanford et al., 2013), but their prediction power is limited to the conditions adequately close to the corresponding steady state.

As a better alternative to approximative kinetics, an approach was established and utilized based on the concept of parametric Jacobian, which covers the behavior of all possible kinetic models that are consistent with an experimentally observed operating point (Steuer et al., 2006). This approach provides an opportunity to detect and analyze bifurcation characteristics of the metabolic network without the need for explicit determination of kinetic parameters. Ensemble modeling of metabolic networks (Tran et al., 2008) is an elegant idea for large-scale kinetic modeling of biochemical reaction networks. In this method, each enzymatic reaction is broken down to its elementary reactions that all follow mass-action kinetics. An ensemble of thermodynamically consistent kinetic models with different dynamic behavior that all converge to a reference steady state is collected with the help of intracellular metabolome data. This ensemble is then filtered by the results of perturbation experiments to filter out inconsistent models from the ensemble and to increase the predictability of remaining models. The approach was successfully applied, among others, to construct kinetic models of *E. coli* (Khodayari et al., 2014) and cancer metabolisms (Khazaei et al., 2012), leading to promising flux predictions.

## Top-Down Approaches to Discover Condition-Specific Metabolic Networks

Time series of metabolite concentrations in response to a perturbation, and also replicates of metabolome data at a specific steady state, both implicitly contain information on the structure of active metabolic network. Reverse engineering of these data to infer the condition-specific metabolic network without necessarily prior knowledge on the genome of the organism and its static metabolic network is an alternative to all bottom-up approaches that are based on the availability of a large-scale stoichiometric model of the organism. Although promising, less attention has been paid to these top-down approaches compared to bottom-ups mainly because of the technical obstacles in gathering reliable metabolome data in large scale. This limitation will be removed with future advancements in the detection and quantification of intracellular metabolites such as higher coverage and temporal resolution. At this stage, however, several research groups have established algorithms and methods for reverse engineering of metabolic networks by using either time series or steady-state replicates of metabolite concentrations (Crampin et al., 2004; Chou and Voit, 2009; Hendrickx et al., 2011; Lecca and Priami, 2013).

### Network discovery based on time-series data

The use of time-series metabolite concentration data to predict the underlying network connectivity information first appeared in the literature about two decades ago. Time-lagged correlations combined with a projection technique called multidimensional scaling were shown to construct the structure of generic biochemical networks with few nodes (Arkin and Ross, 1995). Correlation between time-series profiles of metabolites, with the consideration of the delay in the influence of one metabolite on the next, is the basis of the time-lagged correlation method for the inference of metabolic networks. The approach, called correlation metric construction, was later experimentally verified *in vitro* by inferring the first steps of glycolytic pathway in a 14-metabolite system (Arkin et al., 1997). Modified versions of the approach appeared later (Samoilov et al., 2001; Lecca et al., 2012). In the latter, metabolic pathway of an anticancer drug was deduced from the time-lagged correlations of corresponding metabolite concentration measurements. The modification introduced by the former work was recently improved by using mutual information similarity score rather than simple linear correlation (Villaverde et al., 2014). The authors compared their method, called MIDER, with several other methods by applying it to different types of cellular networks, including *in vitro* glycolytic pathway data. The approach outperformed the other methods.

Another method to reconstitute a network using time-series data is based on perturbation experiments around steady state. The initial curve of concentration changes of metabolites in response to a pulse change on the concentration of a metabolite is processed with the method of zero initial slopes (Vance et al., 2002). The method successfully inferred the structure of glycolysis based on *in vitro* experimental data (Torralba et al., 2003). Performance comparison of the method with the correlation metric construction approach was later provided based on *in silico* data of *S. cerevisiae* and *E. coli* central metabolic networks (Hendrickx et al., 2011). An approach based also on perturbation experiments, but with a different formulation aiming to calculate Jacobian matrix from time derivatives of concentration data, was first applied to gene networks (Schmidt et al., 2005). A modified version of the approach recently used *in vivo* metabolite concentration measurements from tomato seedlings to reconstruct quercetin glycosylation pathway (Astola et al., 2011).

Apart from such model-free structure identification methods, model-based methods use time-series metabolite concentration data not only to identify network structure but also to estimate proper model parameters such as rate constants of kinetic expressions (Chou and Voit, 2009). Majority of these approaches use power-law (also called S-system) formulation (Savageau and Voit, 1987) to approximate reaction kinetics. An approach, for example, used S-system modeling with a multi-objective optimization by simultaneously minimizing the number of interactions and the error in the fitting (Liu and Wang, 2008). They applied their method to major metabolites involved in ethanol fermentation. An earlier work analyzed a small three-metabolite network of phospholipid metabolism by combining S-system modeling and an evolutionary modeling method, genetic programing (Ando et al., 2002). Later, a new representation of S-system approach, called S-trees, was combined with genetic programing to reverse-engineer yeast fermentation pathway in a more efficient manner by using *in silico* time-series concentration data of five metabolites (Cho et al., 2006). In a sophisticated approach, others used symbolic regression based on genetic programing to infer both the structure and the model of yeast glycolytic oscillations from *in silico* data (Schmidt et al., 2011). Their use of acylic graph encoding rather than tree-based encoding together with symbolic regression approach ensured the identification of parsimonious (sparse) models. Rather than S-system formulation, mass-action kinetics can also be used to infer pathway connectivity and reaction mechanism (Srividhya et al., 2007). This minimizes the computational burden on the algorithm since only rate constants are to be estimated as parameters in the mass-action formulation. The authors tested their method with real time course experimental metabolome data of *Lactococcus lactis* glycolysis. A graphical user interface was later made available by the same group to ease the inference of kinetics and network architecture from dynamic data of biochemical pathways (Mourão et al., 2011). Genetic programing was also combined with mass-action kinetics in an algorithm, which ensures the estimation of biochemically more plausible models (Gormley et al., 2013). The small phospholipid network of (Ando et al., 2002) was inferred in a more compact way by this algorithm.

### Network discovery based on steady-state data

The use of steady-state metabolome data to infer metabolic network structure has also drawn attention in the last decade. The biological variability in the metabolism of the organisms at around steady state is a known phenomenon due to slight variations in the enzyme levels or due to slight natural or environment-induced fluctuations within cellular processes. Slight variations in the steady-state measurements of metabolite levels can be informative on the network structure (Steuer et al., 2003; Camacho et al., 2005; Çakır et al., 2009). The most common approach here is to use the similarity measures such as Pearson correlation to assign edges between metabolites. One should note that such correlations are not necessarily strong among neighboring metabolites whereas there could be strong correlations among distant metabolites in the network (Camacho et al., 2005). In a comprehensive study, different alternative similarity measures (linear vs. non-linear, and full vs. partial) were applied to *in silico* metabolome data belonging to two microorganisms to systematically analyze method performances (Çakır et al., 2009). The results revealed no clear superiority between linear (Pearson correlation) and non-linear (mutual information) similarity measures. The best performing method was identified as nth order partial Pearson correlation, known also as graphical Gaussian modeling. Graphical Gaussian modeling was also applied to metabolome data from blood serum samples to reconstruct human fatty acid metabolism (Krumsiek et al., 2011). Others (Nemenman et al., 2007) analyzed *in silico* metabolome data of red blood cell metabolism by ARACNE approach (Margolin et al., 2006), which is based on pruning mutual information scores. An elegant improvement on ARACNE based reverse engineering of metabolic profiling data was suggested later (Bandaru et al., 2011). The approach puts a constraint on the possible metabolic transformations to satisfy the mass conservation between the connected metabolites. Synthetic data covering up to about 200 metabolites were generated to test the approach. One issue in such similarity-based approaches is that only pairwise interactions are aimed to be found. However, a metabolic reaction can involve more than two metabolites. Based on this reasoning, an attempt to also deduce triple interactions by using ternary mutual information was suggested (Diệp et al., 2011). Analysis of synthetic yeast glycolysis data and red blood cell data showed the success of this approach in capturing higher order interactions.

A different approach to discover active metabolic networks from steady-state data is based on Lyapunov equation. In Eq. 1, the rate vector, **v**, is a complex non-linear function of concentrations, **C**. For systems around steady state, the equation can be expressed in terms of Jacobian matrix, **J**, by the help of linear approximation:

(3) $$\frac{d\text{X}}{\mathrm{dt}}\approx \text{JX}$$

with **X** = **C** − **C_s_**, and **C_s_** shows the steady-state metabolite concentrations. Jacobian matrix stores detailed information on the structure of the underlying network; such as the directionality of interaction, strength of interaction, and regulation type of interaction. For small fluctuations around steady state, the right-hand side of Eq. 3 becomes zero, and the left-hand side can be expressed in such a way that a link between the covariance matrix of metabolome data, **Γ**, and Jacobian matrix is provided. The details of the derivation are given elsewhere (Van Kampen, 1992; Steuer et al., 2003).

(4) $$\text{J}\Gamma +\Gamma {\text{J}}^{\text{T}}=-2\text{D}$$

**D** in the equation shows the extent of fluctuations. Eq. 4, known as Lyapunov equation, can be used to infer metabolic network structure since it provides a link between the data-based covariance matrix and network connectivity stored in **J**. Reverse-engineering metabolome data by using the Lyapunov equation was first discussed via a hypothetical three-metabolite system (Steuer et al., 2003). A recent work provided a theoretical analysis on the use of the Lyapunov equation to infer network structure from steady-state metabolome data (Öksüz et al., 2013). The authors used a rearranged version of the Lyapunov equation:

(5) Aj=2d

Here, **j** and **d** are vectorized versions of **J** and **D** matrices. **A** is a matrix based on the covariance of data. In that work, directed networks were inferred from *in silico* metabolome data of *S. cerevisiae* glycolysis, *E. coli* central carbon metabolism, and brain glycolysis by solving Eq. 5 for **j** using a genetic-algorithm based formulation. In the optimization formulation, the dual objective function was simultaneous maximization of the sparse structure and minimization of the residual norm of the equation. When compared to the inference results based on nth order partial Pearson correlation, a much higher prediction accuracy was reported. One other advantage of the optimization-based approach is the fact that Eq. 5 infers a directed network whereas correlation-based approaches cannot predict directions of interactions. The Lyapunov equation was recently used to infer differential changes in Jacobian matrix rather than the inference of network structure by predicting Jacobian matrix itself (Sun and Weckwerth, 2012; Kügler and Yang, 2014; Nägele et al., 2014).

## Paths to Reconcile Bottom-up and Top-Down Metabolic Network Discovery Approaches

Previous sections reviewed bottom-up and top-down metabolic network discovery approaches from literature. Top-down approaches are dependent on intracellular metabolome data, and there are bottom-up approaches, which aim to use omics data as additional constraints. The simultaneous use of both approaches to discover better condition-specific networks has not been a focus in the scientific community. Here, we will elaborate on the ways to reconcile these two approaches when intracellular metabolome data of a condition in question are available.

All model-based top-down approaches using time-series data also infer a Jacobian matrix of the model. Many other top-down approaches are based on correlations between metabolites. There is a significant relationship between the correlation strengths and the strengths of interactions implied by Jacobian entries (Çakır et al., 2009). Therefore, correlation strengths or Jacobian-interaction strengths of the inferred edges can be used as edge scores in the bottom-up constraint-based modeling approaches as additional constraints for a better identification of the active metabolic network as follows: all inferred edges in a top-down approach based on metabolome data are ranked with respect to their edge scores. Afterward, cut-off values for high- and low-scores are determined. If a high-score edge also appears in the corresponding static genome-scale stoichiometric model, that reaction is assigned a high weight. If a high edge-score does not have a corresponding connection in the genome-scale model, this could imply a novel or a regulatory interaction. As it is known, genome-scale metabolic models do not account for regulatory interactions of metabolites with enzymes, however, top-down approaches do not have this limitation since they are purely data-based. If the edge-score is low, the corresponding reaction in the stoichiometric model is assigned a low weight. Similarly, if the top-down approach assigns no edge between two metabolites, which are linked with a reaction in the stoichiometric model, such reactions are also assigned low weight. All other reactions can be assigned with a medium-weight. Then, a mixed-integer programing based optimization framework can be used with Eq. 2 such that the resulting condition-specific flux distribution is as consistent as possible with the edge scores, including maximum possible number of high-weight reactions and minimum possible number of low-weight reactions as active. Thereby, the strength of top-down predictions can be used for better bottom-up flux predictions.

Use of transcriptome or proteome data as constraints in metabolic-flux calculations resulted in several alternative methods such as GIMME, iMAT, and INIT. These approaches remove reactions from the static metabolic reaction set if the controlling gene or protein is not active. However, a recent work comparing all these methods could not identify a method with clear superiority over the parsimonious FBA (Machado and Herrgård, 2014). This approach can be combined with edge scores (inferred Jacobian-interaction strength or calculated correlation strength) information to yield better network identification. GIMME-like approaches remove reactions from the model, this means also removal of metabolites. Two different approaches can be used: (i) removed reactions whose main substrates and products show high edge scores must be retained in the reaction set, implying an active edge (ii) reactions whose main substrates and products show very low and insignificant correlations must be candidates to be removed from the reaction set, implying an inactive edge if their removal does not hamper the objective function. Such a flux calculation powered by the top-down inference of network edges can lead to a more refined network.

One reconciliation approach will be the integrative use of flux-balance equation (Eq. 2) and rearranged Lyapunov equation (Eq. 5). Flux-balance equation was widely used in the last two decades because of its simplicity, requiring only the stoichiometric coefficients of reactions, and few measurement constraints. The rearranged Lyapunov equation bears a similar simplicity since it is only based on the covariances of metabolome measurements. The only major issue, as it is the case in flux-balance equation, is a proper choice of objective function to solve the equation. Since both **J** and **v**, the unknowns in both equations, represent the active network structure, the coupled use of these two equations can be beneficial from two different aspects: (i) a better flux distribution can be found thanks to the metabolome-based constraint provided by Eq. 5, (ii) the information stored in stoichiometric matrix, since it will reveal all possible non-interacting pairs, will provide a constraint to get a better estimate of Jacobian matrix by setting edge scores of some pairs to zero.

An approach getting popular to construct genome-scale kinetic models is ensemble modeling. This modeling approach constructs kinetic models from an ensemble of models, and filters the inconsistent models out by using the results of perturbation experiments (Tran et al., 2008; Khodayari et al., 2014). On the other hand, a number of methods infer networks from time-series data by using a model-based approach. The output of such methods is both the network structure and the dynamic kinetic model with estimated parameters (Srividhya et al., 2007; Liu and Wang, 2008). A number of alternative models are scanned in these methods to infer the most suitable one. Therefore, the strengths of model-based network inference and ensemble-based kinetic model reconstruction can be combined to yield better frameworks.

In summary, both bottom-up and top-down discovery of metabolic networks have come a long way in the last 20 years, providing the scientific community with a number of computational methods, as reviewed in this review. Considering the improvements that are being experienced both on the coverage and precision of metabolome data, the coming decade will witness an exponential increase in the number of metabolome datasets, similar to what was experienced with transcriptome data in the last decade. This review aimed at drawing attention to this point, as ways to reconcile the two major metabolic network discovery approaches will gain increasing importance.

## Conflict of Interest Statement

The authors declare that the research was conducted in the absence of any commercial or financial relationships that could be construed as a potential conflict of interest.
